# A novel nonlinear afterload for ex vivo heart evaluation: Porcine experimental results

**DOI:** 10.1111/aor.14307

**Published:** 2022-05-20

**Authors:** Henry Pigot, Kristian Soltesz, Audrius Paskevicius, Qiuming Liao, Trygve Sjöberg, Stig Steen

**Affiliations:** ^1^ Department of Automatic Control Lund University Lund Sweden; ^2^ Division of Thoracic Surgery, Department of Clinical Sciences Lund University Lund Sweden; ^3^ Department of Cardiothoracic Surgery Skåne University Hospital Lund Sweden

**Keywords:** cardiac afterload, ex vivo heart evaluation, functional evaluation, heart perfusion, heart transplantation, working heart

## Abstract

**Background:**

Existing working heart models for ex vivo functional evaluation of donor hearts often use cardiac afterloads made up of discrete resistive and compliant elements. This approach limits the practicality of independently controlling systolic and diastolic aortic pressure to safely test the heart under multiple loading conditions. We present and investigate a novel afterload concept designed to enable such control.

**Methods:**

Six ∼70 kg pig hearts were evaluated in vivo, then ex vivo in left‐ventricular working mode using the presented afterload. Both in vivo and ex vivo, the hearts were evaluated at two exertion levels: at rest and following a 20 μg adrenaline bolus, while measuring aortic pressure and flow, left ventricular pressure and volume, and left atrial pressure.

**Results:**

The afterload gave aortic pressure waveforms that matched the general shape of the in vivo measurements. A wide range of physiological systolic pressures (93 to 160 mm Hg) and diastolic pressures (73 to 113 mm Hg) were generated by the afterload.

**Conclusions:**

With the presented afterload concept, multiple physiological loading conditions could be tested ex vivo, and compared with the corresponding in vivo data. An additional control loop from the set pressure limits to the measured systolic and diastolic aortic pressure is proposed to address discrepancies observed between the set limits and the measured pressures.

## Graphical abstract

An adaptive cardiac afterload concept is presented for ex vivo heart evaluation that aims to provide safe adjustment of systolic and diastolic pressures to test hearts under a variety of loading conditions. The afterload was tested with six porcine hearts and the results were compared to corresponding in vivo hemodynamic measurements. Across all individuals, the afterload gave physiological systolic and diastolic aortic pressures, and aortic pressure waveforms that matched the general shape of the in vivo measurements.

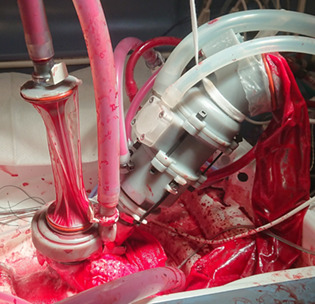

## 1 INTRODUCTION

Ex vivo beating heart models enable the study of denervated heart physiology under varying conditions in physiological isolation. To meet the growing demand for donor heart organs, such models have been investigated as a means to increase the safe use of extended criteria heart donors.^1^ These donors typically include older individuals, those with previous conditions, and donors with circulatory (DCD), as opposed to brain (DBD), determined death.^2^, ^3^ The potential risks of using extended criteria hearts have motivated the study of non‐beating,^4^, ^5^ empty beating,^6^ and working heart models^7^, ^8^, ^9^, ^10^ to perfuse the cardiac muscles and provide indicators of heart performance prior to transplantation.

In contrast to non‐beating and empty beating models, hearts in working mode actively pump perfusate through a flow impedance, referred to as the afterload. The hemodynamic function of the heart can then be observed directly, whereas non‐working models only provide metabolic indicators of heart condition. Although popularized by the first clinically approved ex vivo heart perfusion device,^6^ studies have shown that metabolic metrics are unreliable predictors of post‐transplant outcomes and point to functional metrics as a promising alternative^9^, ^11^, ^12^, ^13^, ^14^, ^15^

An afterload must at least establish the minimum diastolic aortic pressure required for sufficient coronary flow. In order to evaluate a wide range of hearts under a variety of loading conditions, an afterload would ideally enable control of diastolic aortic pressure and systolic aortic pressure, independently of cardiac output, such that they match the needs of the intended recipient patient.

**FIGURE 1:**
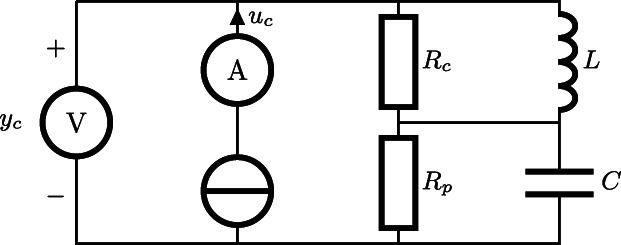
Circuit analogy of the parallel 4‐element Windkessel model with signals: Driving current (aortic flow) *u*_*c*_, corresponding voltage (aortic pressure) *y*_*c*_, and parameters: Peripheral resistance, *R*_*p*_; arterial compliance, *C*; characteristic aortic impedance, *R*_*c*_; and perfusate inertance, *L*.

The systemic arterial tree—the left‐heart afterload in the body—has long been represented using lumped‐parameter linear models known as Windkessel models,^16^ illustrated in Figure 1, to describe the relationship between aortic flow and pressure. Mechanical afterloads have been constructed according to the Windkessel model, with discrete resistive and compliant elements, in an attempt to recreate physiological aortic pressure waveforms.^7^, ^17^, ^18^, ^19^ The peripheral resistance, *R*_*p*_, determines the static gain from flow to pressure, while the compliance, *C*, affects both the systolic and diastolic pressure. Any systolic and diastolic pressure combination can be achieved for a given aortic flow by varying *R*_*p*_ and *C*. However, the parameters are coupled; to adjust only systolic or diastolic pressure, both resistance and compliance must be manipulated. Doing so also changes the shape of the aortic pressure waveform.

Traditionally, ex vivo working heart models using Windkessel‐based afterload designs are constructed with fixed or manually adjustable resistive and compliant elements. Due to the aforementioned coupled parameters, such afterloads are unable to practically emulate a variety of loading conditions and respond to hemodynamic changes in a time‐constrained clinical setting. Two groups have published large‐animal studies on adjustable Windkessel‐based afterloads, though with the exception of Ref. [20] they only tested single loading conditions for each heart.^18^, ^19^ Notably, in Ref. [18] coronary perfusion was controlled with a separate perfusion loop and in Refs. [19, 20] physiological diastolic pressures were not demonstrated. Matching the parameters of discrete Windkessel afterload elements to estimated parameters in a potential recipient has been suggested in Ref. [19]. However, our sensitivity analysis of the Windkessel model with porcine and human data showed that simultaneous identifiability of its parameters from representative data is limited.^21^ This means that parameters identified in vivo under one working condition are not necessarily adequate for evaluation of the same heart ex vivo under another working condition.

As an alternative to Windkessel‐based afterloads, some groups have pumped perfusate retrograde into the aorta with a centrifugal pump to control diastolic aortic pressure.^10^, ^13^, ^20^ These systems ensure diastolic aortic pressure regardless of cardiac output. However, systolic pressure is left uncontrolled and dependent on the geometry and position of the perfusate path and the rotational‐velocity‐dependent flow impedance of the centrifugal pump. Independent control of systolic and diastolic aortic pressures is not practical with this afterload method.

Here we present and investigate a novel afterload concept, designed to allow independent control of hemodynamic parameters critical to the safety and evaluation of the organ under test: diastolic pressure to ensure sufficient coronary flow and systolic pressure to facilitate physiological loading conditions while safely limiting peak aortic pressures. This article is the first evaluation of technology patented in Ref. [22]. To the best of our knowledge, it is the first demonstration of heart evaluation using an adjustable cardiac afterload operating at physiological systolic and diastolic aortic pressures.

The afterload was tested with six hearts from ∼70 kg pigs, representative of adult human hearts, both at rest and in a state of exertion. The objective was to compare observations in vivo and ex vivo for each individual heart to investigate the feasibility of establishing and maintaining a range of physiological loading conditions by adjusting systolic and diastolic aortic pressures. Tight feedback control of the pressures has been left for future work, though a strategy to achieve such control is discussed.

## 2 METHODS

Six ∼70 kg Swedish domestic pigs (*sus scrofa domesticus*) were used in the study. Each heart was evaluated in vivo with an open chest in two states of cardiac exertion: in resting state and in a high‐exertion state induced by a 20 μg adrenaline bolus.

In each state, aortic pressure and flow, ventricular pressure and volume, and atrial pressure were recorded. This was then repeated with each heart in an ex vivo left‐ventricular working heart model using the considered nonlinear afterload, with cardiac output (flow provided to the left atrium), systolic aortic pressure, and diastolic aortic pressure controlled to physiological levels.

Conductance catheter ventricular pressure‐volume signals were recorded at 200 Hz with LabChart 8 (AD Instruments, Boulder, CO). All pressures and aortic flow were sampled with a data acquisition system built in‐house using AD7730 converters (Analog Devices, Norwood, MA) at 200 Hz and low pass filtered with a 50 Hz −3 dB cut‐off frequency—twice the expected frequency of relevant physiological signals. Pulse pressure was calculated as the difference between systolic and diastolic pressure. Instantaneous cardiac power was calculated as the pointwise in time product of left ventricular pressure and aortic flow.

### 2.1 Nonlinear cardiac afterload

**FIGURE 2:**
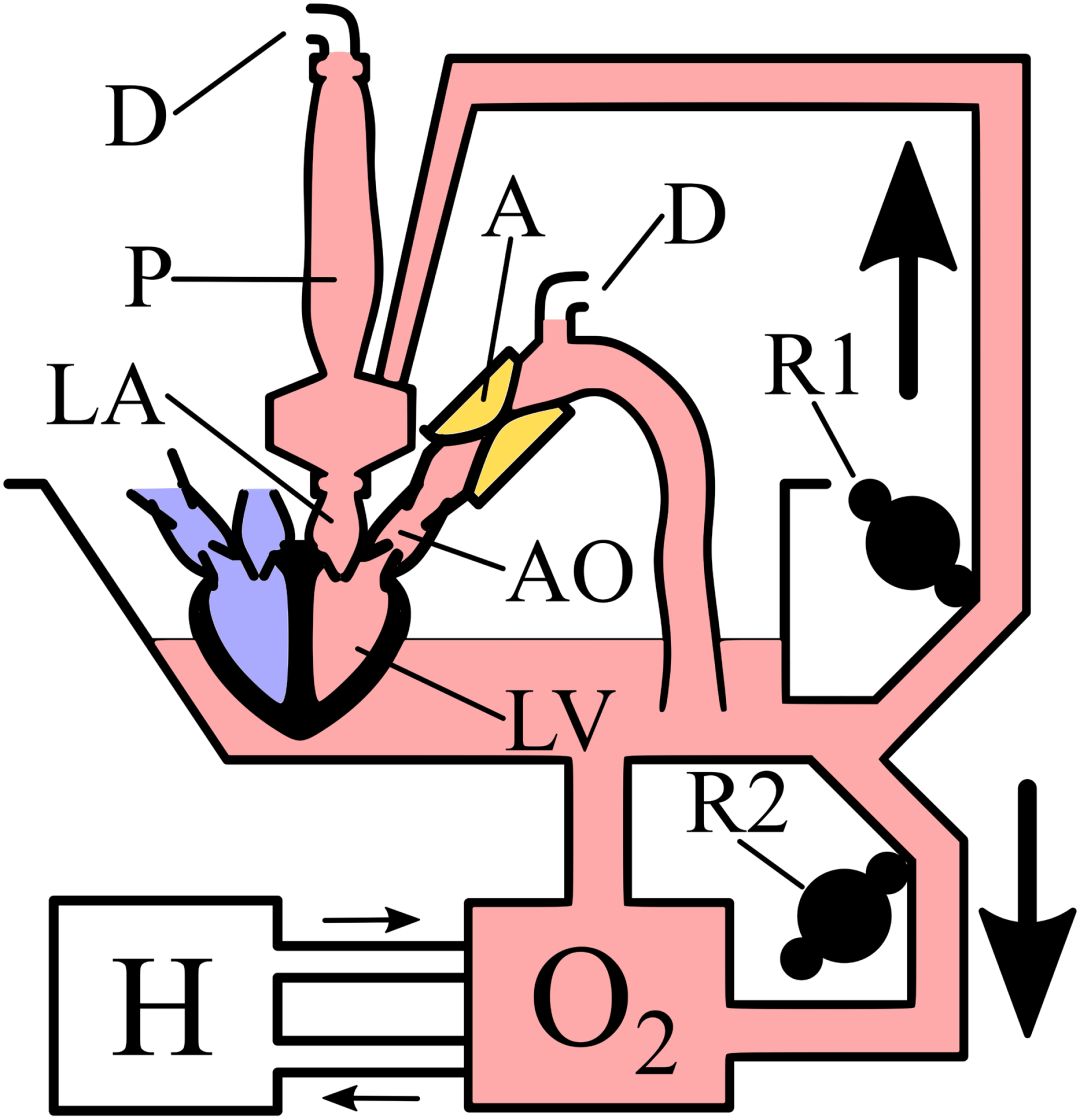
Ex vivo setup, with the afterload (A). Roller pump R1 delivers perfusate to the preload (P) at the left atrium (LA). At the preload, the perfusate goes through a vortex to lower its linear momentum to limit forced atrial filling. A compliant reservoir sits above the vortex, allowing a column of perfusate to accumulate, resulting in atrial preload pressure. Ports D, above the compliant reservoir and the highest point of the afterload, provide de‐airing driven by small roller pumps. The heart pumps the perfusate from the left ventricle (LV) through the aorta (AO) and the afterload and back to the reservoir. The perfusate in the reservoir is circulated by roller pump R2 through an oxygenator (O_2_) that also provides heat exchange via a heater‐cooler unit (H). An additional roller pump (not pictured) is used to provide Langendorff perfusion when the cardioplegic heart is first placed in the system.

**FIGURE 3:**
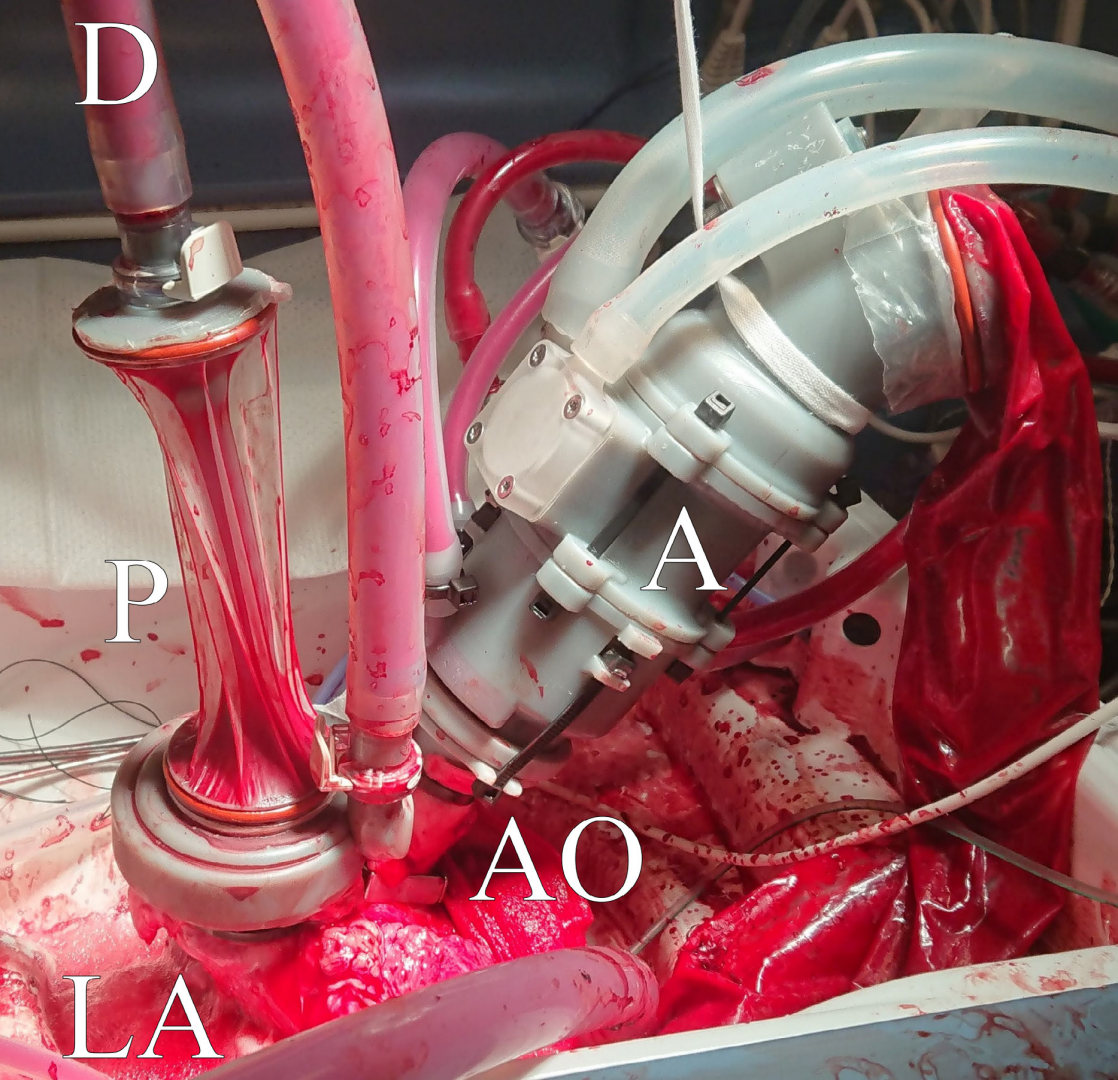
The ex vivo working heart model, with the afterload (A) pictured on the right attached to the aorta (AO), and preload (P) on the left attached to the left atrium (LA) and de‐airing (D). The heart is partially submerged in perfusate.

The ex vivo working heart model is illustrated in Figure 2. The heart is suspended in a perfusate reservoir, with the right atrium at the perfusate surface level. The perfusate is circulated by a roller pump through an oxygenator for heat and gas exchange. A second roller pump delivers perfusate from the reservoir to the left atrium. Above the left atrium, the perfusate first passes through a vortex, pictured on the bottom left in Figure 3. This is not primarily designed to be physiological, but rather to lower perfusate linear momentum and limit forced atrial filling. A compliant sleeve above the vortex allows perfusate to accumulate, generating preload according to the balance between the input flow from the roller pump and the cardiac output. At steady state, roller pump flow to the left atrium is used as a measurement of cardiac output. The heart ejects perfusate from the left ventricle through the adjustable afterload attached to the aorta, generating pulsatile aortic flow. A third roller pump, connected to the aorta, enables Langendorff perfusion of the coronaries arteries—for example, when first attaching a cardioplegic heart to the system or as a safe fallback in the event of heart fibrillation.

**FIGURE 4:**
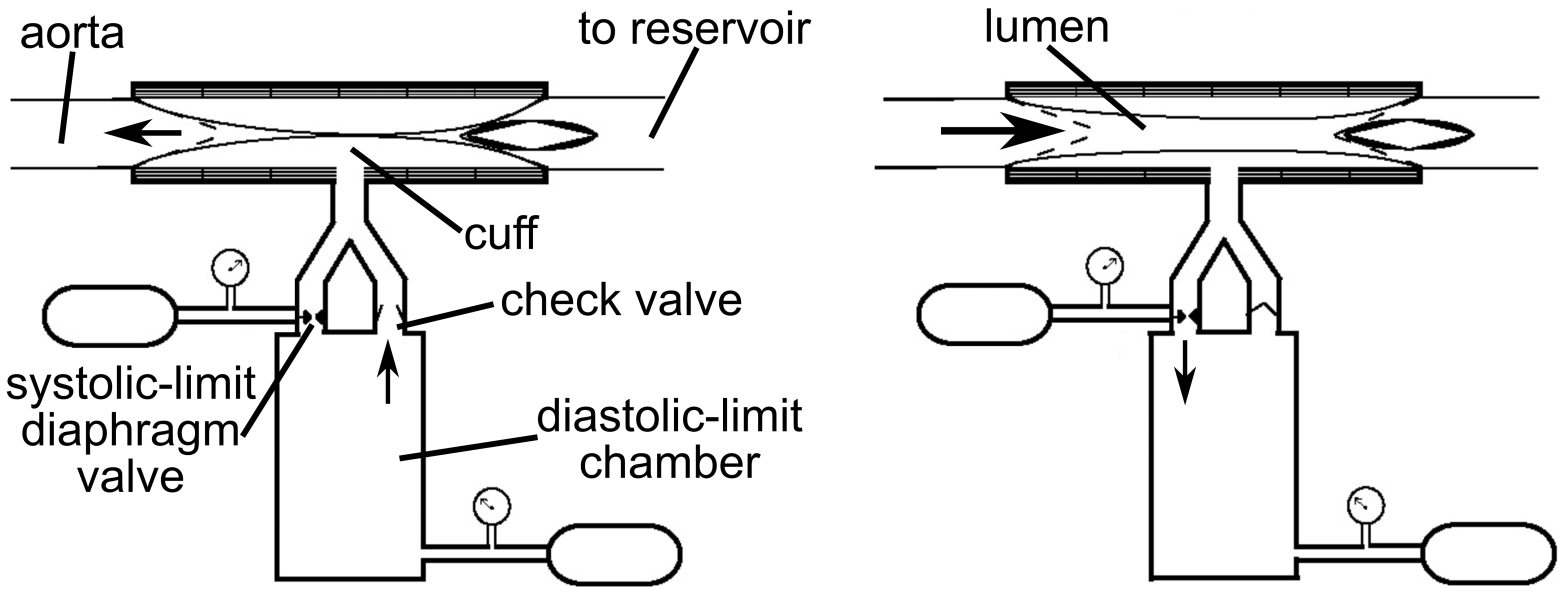
Schematic of the nonlinear adjustable afterload in diastole (left) and systole (right) adapted from Ref. [22]. Black arrows indicate the direction of fluid flow. Two pressures in the afterload are continuously controlled: The pressure in the diastolic‐limit chamber, and the pressure at which the systolic limit diaphragm valve opens. In diastole, air flows through a check valve from the diastolic‐limit chamber into the cuff. The air pressure in the cuff is exerted on the perfusate in the aorta, facilitating coronary perfusion. In systole, the contracting ventricle causes the perfusate pressure in the aorta to rise, compressing the air in the cuff, opening a perfusate lumen through the cuff, and closing the check valve. If the air pressure in the cuff exceeds the set point of the systolic‐limit diaphragm valve, air flows through the valve back into the diastolic‐limit chamber, maintaining the systolic pressure set point in the cuff and widening the lumen through the cuff.

The afterload is a pressurized air‐filled cuff through which the heart forces perfusate, as illustrated in Figure 4. The cuff is made of compliant polyisoprene, surrounded by a rigid plastic shell. The pressure limits of the air in the cuff are continuously controlled to a user‐defined setpoint, corresponding to the diastolic and systolic aortic pressure limits. The minimum (diastolic) pressure limit is enforced by a large pressure‐controlled compliant chamber and a check valve. As the ventricle relaxes any perfusate flowing through the cuff is pressed back into the aorta or out into the reservoir by the pressurized cuff as its lumen closes. If the cuff pressure drops below the diastolic‐limit‐chamber pressure, air flows from the chamber through the check valve into the cuff. The cuff presses against the perfusate in the aorta at the air pressure set in the diastolic‐limit chamber (see the left side of Figure 4), facilitating coronary perfusion.

The maximum (systolic) cuff air pressure limit is set by a pressure‐regulated diaphragm valve. As the ventricle contracts, the perfusate exerts pressure against the cuff; the air in the cuff is compressed and its pressure rises, closing the check valve and opening a lumen through which perfusate flows back to the reservoir. If the air pressure in the cuff exceeds the systolic‐limit setting, the diaphragm valve opens and air escapes back into the diastolic‐limit chamber, widening the lumen to limit perfusate pressure in the aorta (see the right side of Figure 4). This nonlinear variation of flow impedance as a function of pressure is designed to increase evaluation safety ex vivo, where the heart lacks protection from over‐distention that is provided by the pericardium in vivo.

### 2.2 In vivo evaluation

All animals were treated according to European guidelines,^23^ under ethics approval 5.8.18–15906/2020 issued by “Malmö/Lunds Djurförsöksetiska Nämnd” (local REB). Porcine experiments were motivated by the physiological similarity between porcine and adult human hearts, and to accurately test the afterload in the absence of realistic heart models.

Sedation was induced with an intramuscular bolus of 1 g ketamine (Ketaminol vet, Intervet, Boxmeer, Netherlands), 140 mg xylazin (Rompun vet, Bayer AB, Solna, Sweden), and 750 μg atropine (Atropin, Mylan AB, Stockholm). Anesthesia was induced with an intravenous bolus of 100 μg fentanyl (Fentanyl, B. Braun Melsungen AG, Danderyd, Sweden) and 20 mg midazolam (Midazolam accord, Accord healthcare Ltd. United Kingdom). An additional intravenous bolus of 40 mg rocuronium (Rocuronium, Fresenius Kabi, Graz, Austria) was given pre‐tracheotomy. Anesthesia was maintained with 12 to 15 ml/h continuous intravenous infusion of the following drugs mixed into a 50 ml syringe: 10 ml of 100 mg/ml ketamine; 6 ml of 5 mg/ml midazolam; 20 ml of 10 mg/ml rocuronium; and 14 ml of 0.9% NaCl saline solution. Normoventilation (PaCO_2_ around 5 kPa), was obtained using a tidal volume of 8 ml/kg body weight at about 20 breaths/min, and a positive end‐expiratory pressure of 5 cmH_2_O.

Separate catheters (Secalon‐T, Merit Medical, Singapore) were inserted into the right atrium via the right internal jugular vein for pressure measurement and anesthesia maintenance, as well into the aorta via the right carotid artery for pressure measurement and blood gas sampling. Heparin was administered (400 U/kg). Median sternotomy was performed. A pressure‐volume catheter (VentriCath 510S, Millar Inc, Houston, TX) was inserted into the left ventricle via the ascending aorta and the left atrium was catheterized for pressure measurement. Non‐ventricular pressures were measured with Meritrans DTXPlus transducers (Merit Medical, Singapore). Aortic flow was measured with an ultrasonic transit‐time flow probe (20PS, Transonic Systems Inc, Ithica, NY). The probe was calibrated against the roller pump supplying perfusate to the left atrium ex vivo, using simultaneous probe and roller pump time‐volume measurements collected prior to the start of experiments. This aortic flow was used as a measurement of in vivo cardiac output. Blood gas measurements (ABL 700, Radiometer, Copenhagen, Denmark) were taken prior to at‐rest measurements to ensure normal blood chemistry. In vivo hemodynamic measurements were recorded at rest and following a 20 μg adrenaline bolus.

### 2.3 Ex vivo evaluation

The ex vivo system was primed with perfusate composed of 1.5 L Krebs solution with 5% Dextran 40, and 7% albumin, into which fresh whole blood from the donor pig was mixed to achieve a mean hematocrit of 22% (2% standard error, hereafter SE), making a total volume of ∼3 L. The oxygenator kept the perfusate at normothermia and facilitated gas exchange using 100 ml/min of 95% oxygen and 5% carbon dioxide. In addition to the heparin left in the whole blood, 5000 U were added to the perfusate. Pharmacological support for heart function in the absence of the pituitary gland and brain stem was provided via continuous infusion, as specified in Ref. [24]: 1 mg adrenaline and 1 mg noradrenaline for vascular tonus, heart rate, and heart contractility, 1 mg cocaine to prevent noradrenaline reuptake, 0.3 mg triiodothyronine, and 300 mg cortisol were all diluted with 0.9% NaCl saline solution into a 50 ml syringe. Infusion rates were initially set at 0.1 ml/h and adjusted up to 4 ml/h according to need.

After the in vivo testing, the heart was preserved with St. Thomas' cardioplegic solution at 4°C. Custom‐made cannulas were fastened to the aorta and left atrium. The pressure‐volume‐loop catheter was inserted into the left ventricle via the aorta, and the left atrium was cannulated for pressure measurement. The cardioplegic time was 30 min (4 min SE).

The heart was mounted into the system, connecting the preload and afterload to the atrium and aorta, respectively. To de‐air the system, the aorta was positioned vertically, then the heart was slowly filled with perfusate via the left atrium, with the afterload set fully open (both pressure limits set to 0 mm Hg). Flow to the atrium was then stopped, and the heart was flushed by pumping perfusate into the aorta at 900 ml/min and setting the afterload diastolic‐limit‐chamber pressure to achieve a mean aortic pressure of 50 mm Hg, leaving the systolic‐limit pressure set to 0 mm Hg. This provided pressure‐regulated coronary flow (Langendorff perfusion) to flush and warm the heart. Perfusate not flowing into the coronary arteries escaped through the afterload to the reservoir. Defibrillation was provided in the event of ventricular fibrillation. Once empty‐beating sinus rhythm was established, the pump to the aorta was stopped and perfusate was provided to the left atrium via the preload, initiating working mode perfusion.

Cardiac output (roller pump flow to the left atrium), diastolic‐limit‐chamber pressure, and systolic‐limit pressure were then slowly adjusted to physiological resting levels, aiming to match the heart's measured in vivo values. However, its observed performance ex vivo was considered when adjusting, so as not to damage the heart in an attempt to perfectly match the in vivo loading. Measurements were taken after the heart reached a steady state. The system was similarly readjusted after administering the 20 μg adrenaline bolus.

## 3 RESULTS

**FIGURE 5:**
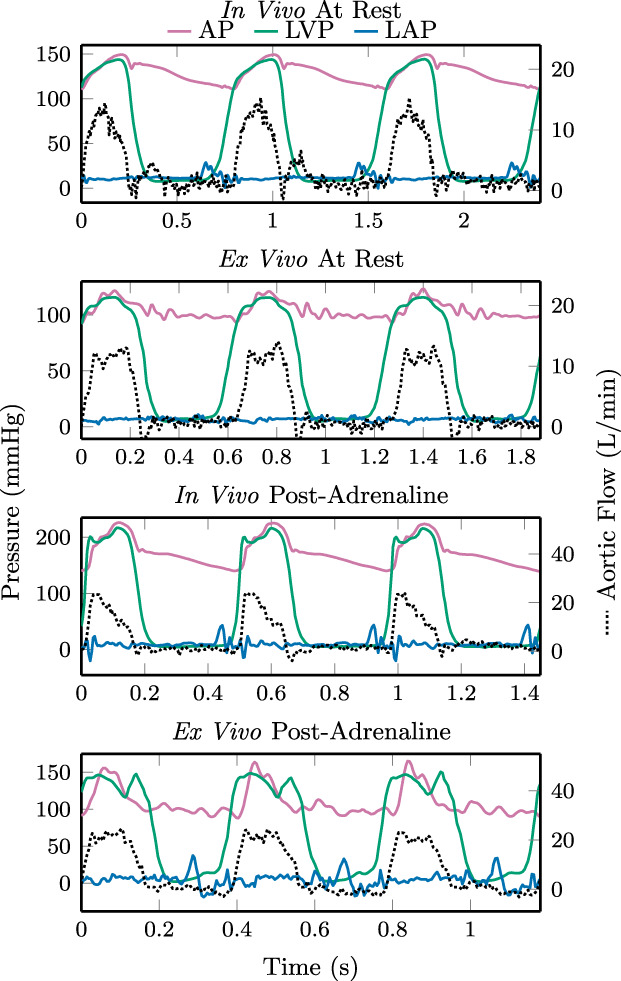
Comparison of aortic pressure (AP), left ventricular pressure (LVP), left atrial pressure (LAP), and aortic flow in vivo and ex vivo, at rest and after a 20 μg adrenaline bolus. Measurements are from Heart 1 and representative of the group.

**FIGURE 6:**
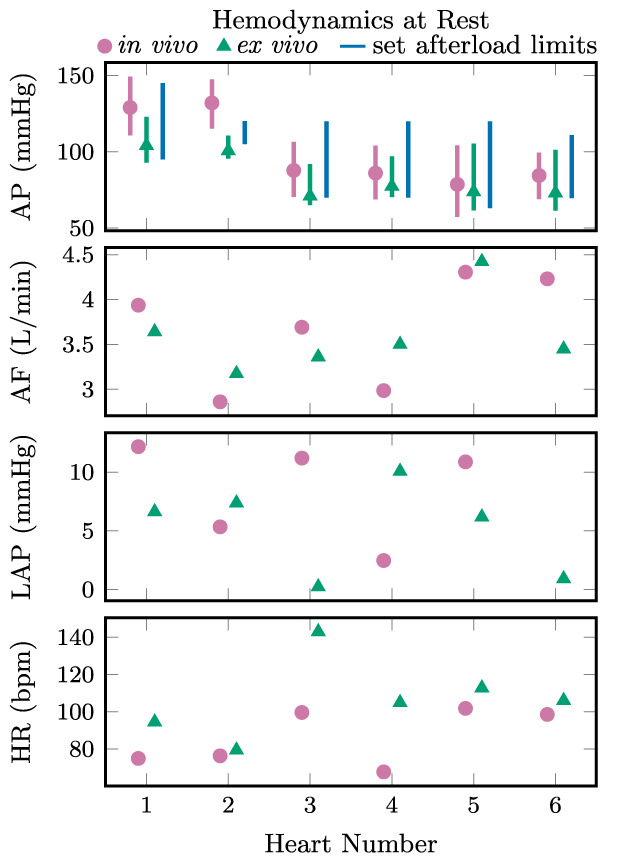
Aortic pressure (AP), aortic flow (AF), left atrial pressure (LAP), and heart rate (HR) in vivo (pink, circles) and ex vivo (measured values in green with triangle markers, interval between set afterload limits in blue) for each heart at rest. The pink and green bars show the total range and the markers show the mean of each value averaged over five cardiac cycles.

**FIGURE 7:**
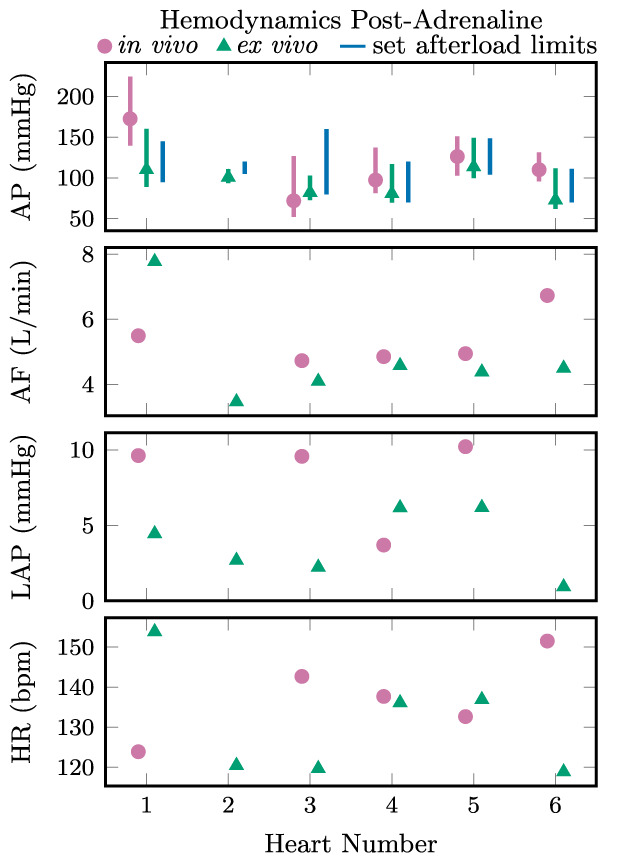
Aortic pressure (AP), aortic flow (AF), left atrial pressure (LAP), and heart rate (HR) in vivo (pink, circles) and ex vivo (measured values in green with triangle markers, interval between set afterload limits in blue) for each heart after a 20 μg adrenaline bolus. The pink and green bars show the total range and the markers show the mean of each value averaged over five cardiac cycles.

Cardiac cycles representative of the group is shown in Figure 5. In vivo and ex vivo hemodynamics are shown for each heart at rest in Figure 6 and after a 20 μg adrenaline bolus in Figure 7. Aortic pressures are shown with the corresponding afterload limit settings, with the mean (markers) and total range (bars, representing systolic and diastolic pressures) averaged over five representative cardiac cycles. Similarly, the mean aortic flow, left atrial pressure, and heart rate are shown. Across all hearts ex vivo, these mean systolic and diastolic pressures had ranges 93 to 123 mm Hg and 73 to 104 mm Hg, respectively, at rest, and 103 to 160 mm Hg and 73 to 113 mm Hg, respectively, post‐adrenaline. Grouped by exertion level, mean systolic, diastolic, and pulse pressures were slightly higher in vivo than ex vivo over five representative cardiac cycles: +14 (SE 6) mm Hg, +6 (SE 4), and +7 (SE 4) mm Hg, respectively, at rest (*n* = 6), and +26 (SE 10), +16 (SE 12), and +10 (SE 10) mm Hg, respectively, post‐adrenaline (*n* = 5).

In four of six hearts, blood loss during in vivo cannulation led to hypovolemia, requiring saline infusion and, in the case of Heart 6, defibrillation and continuous adrenaline infusion (0.1 μg/kg/min). Heart 6 received excessive defibrillation energy in vivo due to a defibrillator error, after which it was frequently arrhythmic. As a result, the left atrium in Heart 6 was not catheterized for pressure measurement in vivo, and the heart showed little response to adrenaline ex vivo, so the diastolic and systolic afterload limits were not raised from the resting level. Heart 2 had chronic pericarditis resulting in poor left ventricular performance and making it prone to arrhythmia. As such, an adrenaline bolus was not administered to this individual ex vivo. Despite the instability of these hearts, they were successfully perfused in working mode at physiological systolic and diastolic aortic pressures.

**FIGURE 8:**
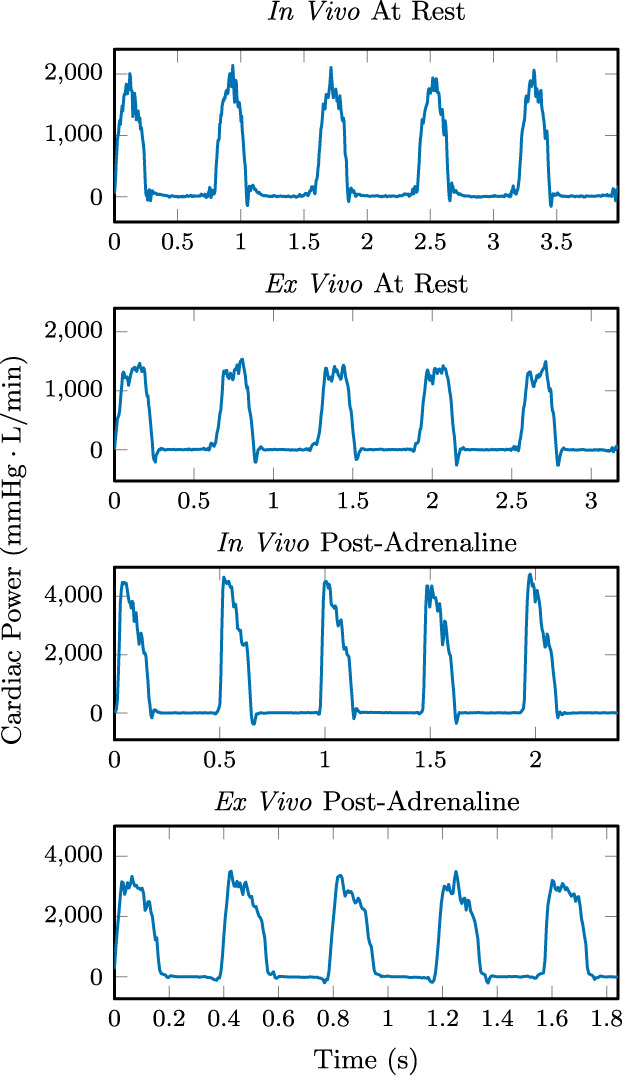
Instantaneous cardiac power of Heart 1, calculated as the pointwise in time product of left ventricular pressure and aortic flow, at rest and after a 20 μg adrenaline bolus, both in vivo and ex vivo.

Figure 8 shows instantaneous cardiac power (CP) representative of the hearts tested. Across all individuals, there was no significant difference in mean CP in vivo and ex vivo, while peak cardiac powers were significantly higher in vivo in particular in the post‐adrenaline case. The differences between in vivo and ex vivo mean CP were +26 (SE 19) mm HgL/min at rest (*n* = 6), and +125 (SE 83) mm HgL/min at post‐adrenaline (*n* = 5), while the differences in peak CP were +384 (SE 109) mm HgL/min at rest (*n* = 6), and +1550 (SE 196) mm HgL/min at post‐adrenaline (*n* = 5).

The waveforms in Figures 5 and 8 are in systole for a larger portion of each cardiac cycle ex vivo compared to in vivo, as is expected due to the higher ex vivo heart rates. Shorter ventricular filling times at these ex vivo heart rates may account for lower peak CP.

3.1

**FIGURE 9:**
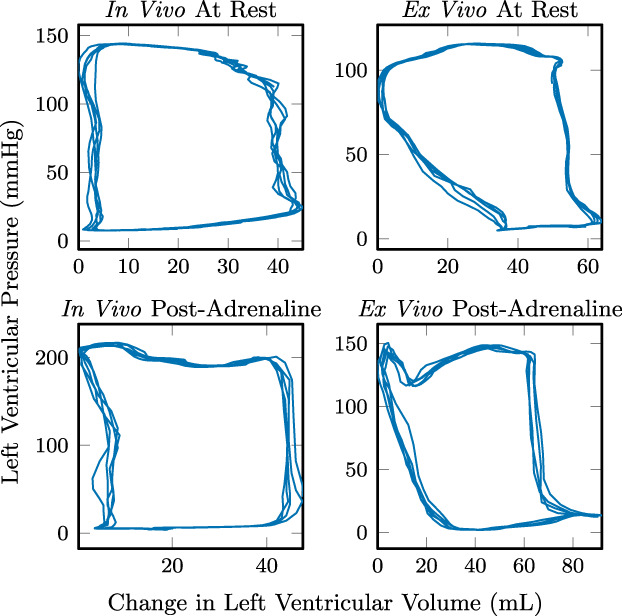
Left ventricular pressure‐volume loop measurements over five cardiac cycles in Heart 1.

Difficulties in the placement and orientation of the conductance catheter resulted in unreliable volume measurements. With the exception of three in vivo and four ex vivo treatments out of a total of 23, the volume measurements were contrary to the physiological pressure and flow waveforms measured. Figure 9 shows the one heart where all four treatments gave representative pressure‐volume loops. The ex vivo loops show early filling of the ventricle during diastole. The ex vivo heart is susceptible to aortic valve insufficiency; early filling is observed in previously published ex vivo working heart pressure‐volume loops using various afterloads.^13^, ^19^, ^20^ The pressure peak at end‐systole ex vivo post‐adrenaline is suspected to be a measurement artifact caused by compression of the pressure transducer against the ventricle wall.

## 4 DISCUSSION

We have evaluated a novel ex vivo cardiac afterload with independently adjustable systolic and diastolic pressure limits. It was tested with six porcine hearts at rest and following an adrenaline bolus, with systolic and diastolic aortic pressure and cardiac output adjusted to physiological levels in both cases. The afterload generated multiple loading conditions in each heart representative of physiological values, though yielding slightly lower ex vivo pressures as compared to in vivo pressures for the same heart. The general shape of the ex vivo aortic pressure waveforms matches the in vivo measurements, as seen in Figure 5. As this study is an initial evaluation of the afterload concept, data were collected from only six hearts. Given this low number, statistical analysis beyond means and standard errors has been omitted.

A discrepancy between the afterload limit values and the observed systolic and diastolic aortic pressures is seen in Figures 6 and 7. The column of perfusate between the afterload cuff and the aortic pressure transducer contributes a positive offset between the aortic pressures and their corresponding limits in the afterload. Although the afterload is designed to behave in an on–off fashion at the systolic and diastolic pressure limits, the air volume in the cuff contributes compliance, and the lumen through the cuff contributes resistance. As a result of this resistive property, increasing flows yield slightly higher systolic pressures even at the same afterload pressure limits, for example during the onset of increased heart rate and higher peak aortic flows post‐adrenaline, so long as the pressures remain below the set limit. An exception is seen in Heart 1 post‐adrenaline, where systolic aortic pressures exceed the set limit. Occasional sticking of the systolic limit diaphragm valve was observed, and in this case the valve may have not opened fully, limiting the displacement of volume from the cuff, and preventing a widening of the afterload lumen.

**FIGURE 10:**
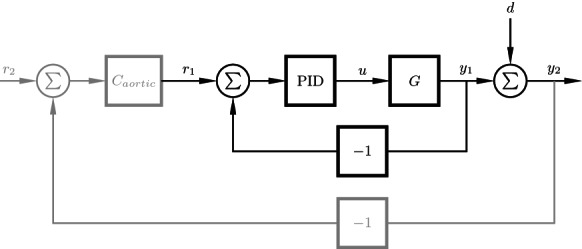
The feedback control structure used for the diastolic‐ and systolic‐limit pressures in the afterload, with cuff dynamics *G* and a hand‐tuned PID controller. In the diastolic case, the pressure of the diastolic‐limit chamber is controlled (setpoint *r*_1_ and measurement *y*_1_), with a roller pump forcing air into the chamber at flow rate *u*. In the systolic case, a roller pump generates air flow rate *u* to pressurize the membrane of a diaphragm valve; the pressure in the cuff must exceed the set pressure of the diaphragm valve (setpoint *r*_1_ and measurement *y*_1_) to escape from the cuff back into the diastolic‐limit chamber. Disturbance *d* represents the offset between the measured diastolic or systolic aortic pressure and the corresponding afterload pressure. An outer loop (gray) may be added to compensate for *d* using aortic pressure feedback, with diastolic or systolic‐limit set point *r*_2_ and corresponding aortic measurement *y*_2_.

When the afterload is operating in pressure ranges between the diastolic and systolic limits, there are two possible mechanisms for volume displacement in the cuff, allowing the lumen through the afterload to widen. Cuff volume can be reduced by compression of the air in the cuff, or by leakage through the valves. An ideal systolic‐limit diaphragm valve would not allow any airflow out of the cuff until the limit pressure is exceeded. Consequently, increases in the systolic‐limit pressure would have no impact on the observed systolic aortic pressure while the heart is generating pressures below the set limit. However, this was not the behavior observed. At systolic aortic pressures below the limit, an increase in the systolic limit pressure resulted in increased systolic aortic pressure. Rather than behaving in an on–off fashion, the systolic‐limit diaphragm valve acts as a variable resistance with the systolic limit controlling the resistance of air leakage from the cuff through the valve. As a result, the systolic aortic pressure is controllable via the systolic limit despite being below the set limit. Furthermore, when operating between the set limits, systolic aortic pressure depends on the diastolic pressure limit, since both air compression in the cuff and leakage through the systolic‐limit diaphragm valve are proportional to the difference between cuff pressure and the diastolic‐limit pressure. Managing this coupled behavior manually becomes impractical in a time‐pressured clinical setting. Instead, we propose an additional control loop to set the pressure limits in the afterload according to feedback of the measured systolic and diastolic aortic pressures, as illustrated in gray in Figure 10. This would enable more rapid and accurate control of the loading conditions while maintaining the safety limits offered by the nonlinear behavior of the adjustable afterload. To similarly maintain safe beat‐to‐beat systolic and diastolic aortic pressures would require impractically fast parameter adjustment in a Windkessel‐style afterload. An example of manually implemented cascaded control was done with Heart 5, matching ex vivo aortic pressures to those measured in vivo, as shown in Figures 6 and 7.

## 5 CONCLUSION

The afterload demonstrated the ability to recreate a variety of cardiac loading conditions ex vivo, under varying levels of exertion and across multiple hearts. The afterload concept enables control of diastolic and systolic aortic pressure by means of the air cuff pressure limits. However, slight discrepancies between the set pressure limits and resulting aortic pressures were observed. While possible to manually compensate for these, as done with Heart 5, doing so in a future clinical setting is impractical, and a cascaded automatic feedback control structure is therefore proposed.

## AUTHOR CONTRIBUTIONS

Henry Pigot wrote the manuscript. All authors reviewed and edited the manuscript. Additionally, Pigot carried out conceptualization, data curation, formal analysis, investigation, software, and visualization. Soltesz contributed to conceptualization and funding acquisition. Paskevicius contributed to investigation, methodology, and software. Liao carried out investigation and contributed resources. Sjöberg was involved in project administration and resources. Steen contributed to conceptualization, funding acquisition, investigation, methodology, and supervision.

## CONFLICT OF INTEREST

The authors declare no conflicts of interest.
